# Supplementary material for: Dirac surface states and nature of superconductivity in Noncentrosymmetric BiPd
Source: Nat Commun. 2015 Mar 30;6:6633. doi: 10.1038/ncomms7633 (PMC4389226; doi:10.1038/ncomms7633)
Supplement: Supplementary Information — Supplementary Figures 1-5, Supplementary Notes 1-3 and Supplementary References [file ncomms7633-s1.pdf]

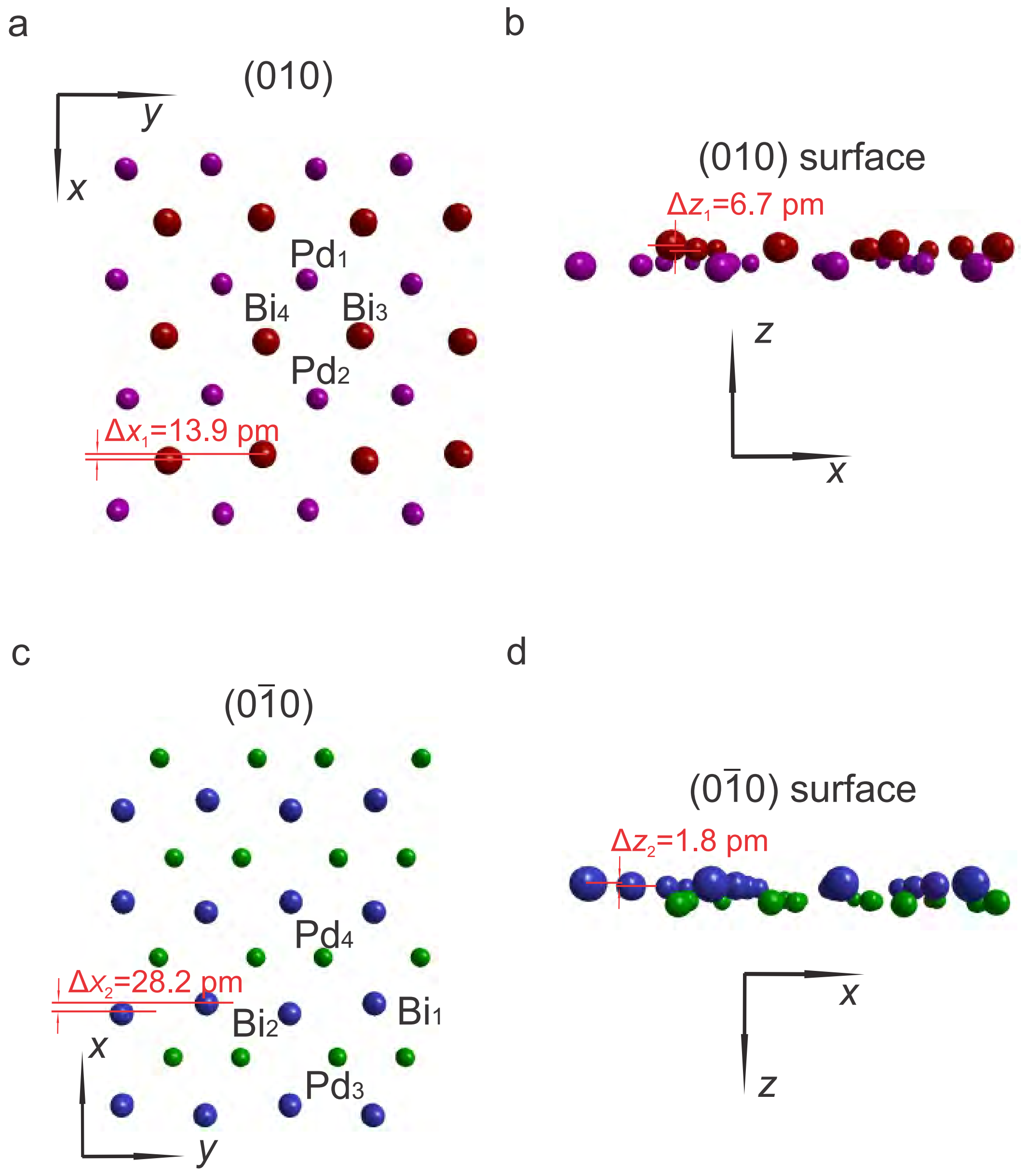

Supplementary Figure 1. Surface Terminations of α-BiPd. Expected surface terminations for a Bi-terminated surface. (**a**, **b**) (010) termination, in **a** shown from top and **b** in a side view. (**c**, **d**) (0$\bar{1}$0) termination, (**c**) shows a top view and (**d**) a side view. It becomes apparent that for the (0$\bar{1}$0) termination, the lateral displacement of Bi atoms is larger, whereas for the (010) termination, the vertical buckling is dominant. The drawn structures are assuming no surface relaxation and are based on the crystal structure shown in ref. 1.

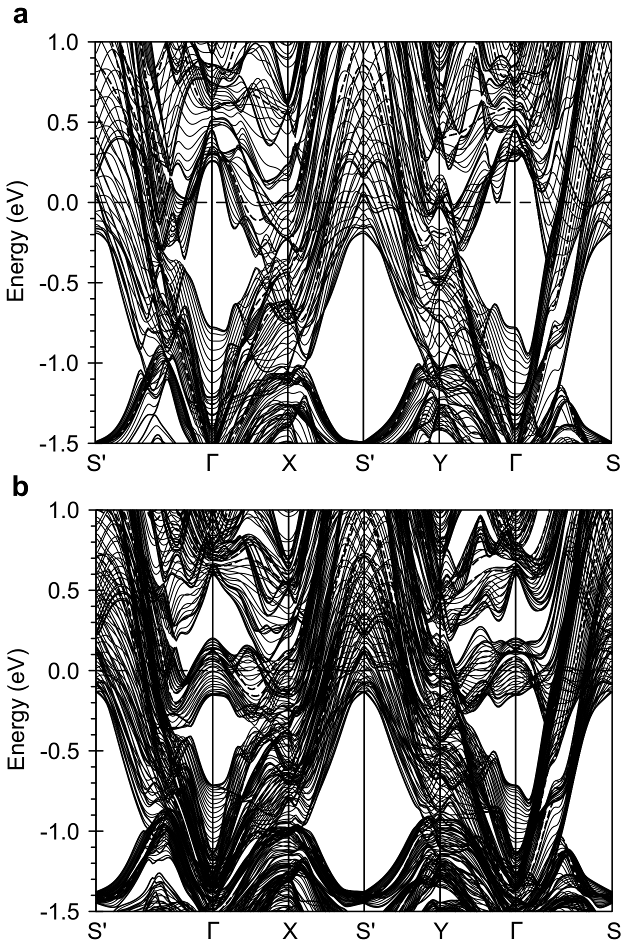

Supplementary Figure 2. Bulk band structure of BiPd projected onto the (010) surface (**a**) without spin-orbit interaction and (**b**) with spin-orbit interaction.

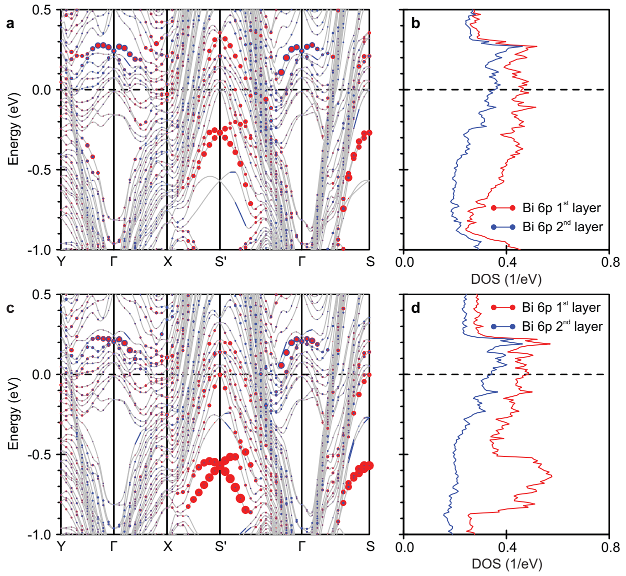

Supplementary Figure 3. Surface band structure. The size of the filled circles is proportional to the weight of Bi 6p states of the first and second outermost layers. (**a**) projected electronic structure and (**b**) surface density of states for the (010) termination; (**c**), (**d**) for the $(0\bar{1}0)$ surface.

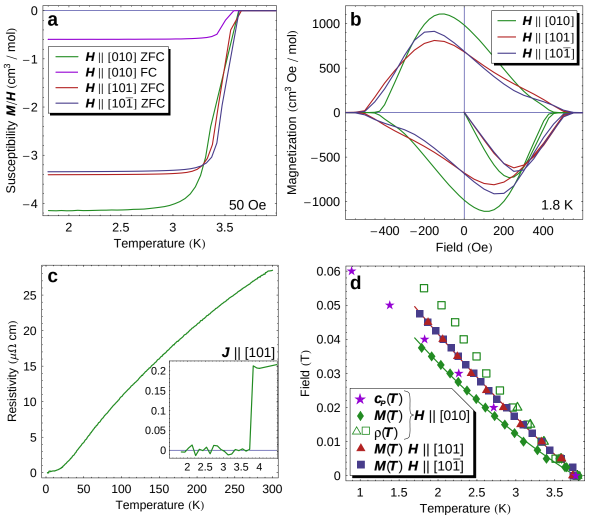

Supplementary Figure 4. Physical Characterization of α-BiPd. (**a**) Magnetic susceptibility as a function of temperature, showing the superconducting transition at ≈ 3.6 K in a field of 50 Oe. (**b**) Magnetization ($M - H$) loop indicating bulk superconductivity with an H_{c2} of ~ 50 mT. (**c**) The zero-field resistivity at a high drive current of 5 mA, which is in good agreement with the one reported previously [2]. (**d**) $H - T$ phase diagram constructed from magnetization and resistivity, showing the upper critical field H_{c2} as a function of temperature.

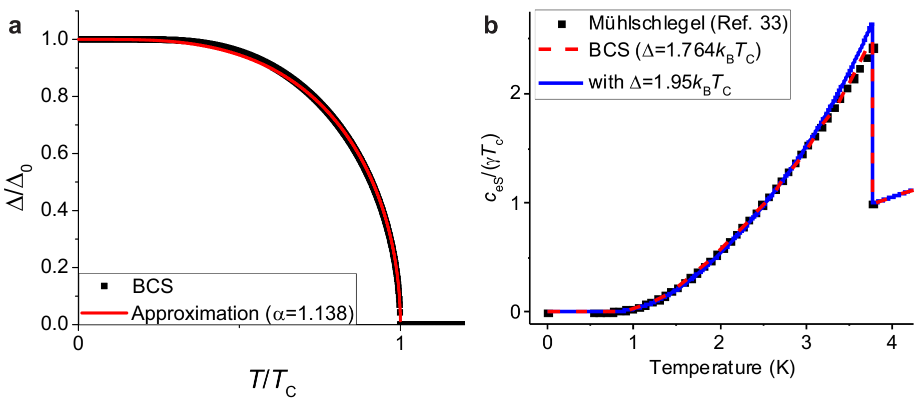

Supplementary Figure 5. (**a**) $\Delta(T)$ vs. T obtained from the BCS gap equation (points) and from $\Delta(T) = \Delta_0 \cdot \tanh\left(\alpha\frac{\pi}{2} \cdot \sqrt{\frac{T_c}{T} - 1}\right)$. ((**b**) Specific heat obtained from BCS theory as tabulated in Ref. 3 (solid squares), from Eq. (1) with $\Delta_0 = 1.764 k_B T_c$ (red dashed line) and with $\Delta_0 = 1.9 k_B T_c$ (blue solid line). γ is the linear coefficient of the electronic contribution to the normal state specific heat, i.e. $C_{en} = \gamma T$.

Supplementary Note 1. Electronic-structure calculations

To assess the influence of spin-orbit coupling on the electronic structure in the bulk, we have performed fully relativistic *ab-initio* calculations. Supplementary Figure 2 displays the bulk energy-band dispersions of BiPd projected onto the (010) surface along high symmetry lines of the surface Brillouin zone of Fig. 2f (see main text). Spin-orbit interactions lead to a spin-splitting as well as to a shift of the bands in energy [compare Supplementary Figure 2a with Supplementary Figure 2b]. With increasing spin-orbit interaction the $p_{1/2}$ bands at the Γ point move down in energy, opening up a band gap within which a Dirac-cone surface state appears, see Figure 2 of the main text and Supplementary Figure 3. We find that around the Fermi energy E_F, the bands are mainly of Bi $6p$ orbital character with subdominant but non-negligible contributions of Pd $4d$ states. Thirteen bands cross E_F leading to several disconnected Fermi surfaces, some of which are quasi two-dimensional with cylindrical shapes oriented along the k_z direction of the Brillouin zone (BZ) (cf. Fig. 2e, f). The strong atomic SOC of Bi induces a spin splitting of the bands of the order of tens of meV and, moreover, results in a large energy shift of states that have predominant $p_{1/2}$ orbital character [4] and moves them into a directional band gap near the Γ-point (see Supplementary Figure 2).

In Supplementary Figure 3 we present the momentum-resolved surface density of states at the two Bi-terminated sides of BiPd. This figure should be compared with Fig. 2 in the main text.

Supplementary Note 2. Sample Characterization

The crystals were characterized by magnetization measurements in magnetic property measurement systems (MPMS-7 and MPMS-XL by Quantum Design) with the reciprocating sample option (RSO), and resistivity was measured in a Physical Property Measurement System (PPMS, by Quantum Design) by a standard four-wire technique, with a high drive current of 5 mA — the absolute value may not be intrinsic due to possible cracking in the sample or scattering at twin boundaries, and the accuracy is limited by estimates of the sample size.

The resistivity data in Fig. 3a were measured on a bar-shaped sample of size $0.33 \times 0.23 \times 1.1$ mm^3 using low temperature transformers to improve the signal to noise. The measure-

ment current was 0.5mA. The bar was prepared by cutting a piece from the ingot using a wire saw and then mechanically cleaving this under liquid nitrogen, to give a bar with cleaved (010) or ($0\bar{1}0$) faces on the top and bottom; the 0.23 mm dimension is the thickness perpendicular to these faces.

The superconducting transition temperature has been extracted from magnetometry, transport and specific heat experiments. The samples have also been characterized by X-ray diffraction to confirm the crystal structure.

As can be seen from Supplementary Figure 4, single crystals of α-BiPd have a $T_c \sim 3.8$ K as determined from magnetic susceptibility and transport measurements. Magnetization loops with magnetic field applied along different crystallographic directions taken at 1.8 K yield an upper critical field around 40 to 60 mT at 1.8 K, with the smallest value for $H \parallel b$. With the exception of the magnitude of the upper critical field H_{c2} (extracted from the midpoint of the resistive transition and where the field-cooled magnetization reached 10% of its full, low-temperature, low-field value), our data agree well with those published previously [2], including our residual resistivity ratio of up to 140. For H_{c2}, our results are consistent with previously published SQUID measurements [5], though somewhat lower than what has been reported from transport [2]. Our resistivity data only agree with other techniques when high drive currents are used.

Supplementary Note 3. Specific Heat Calculation

The electronic contribution to the specific heat has been calculated from

$$C_{es} = \int_{-\infty}^{\infty} f'(\epsilon)\rho(\epsilon)\left(\frac{\mathrm{d}\Delta(T)^2}{\mathrm{d}T} - \frac{2}{T}\epsilon^2\right)\mathrm{d}\epsilon, \quad (1)$$

with $E(k)^2 = \epsilon_k^2 + \Delta(k)^2$, $\rho(\epsilon)$ the superconducting density of states (DOS), $\Delta(T) = \Delta_0 \cdot \tanh\left(\alpha\frac{\pi}{2}\cdot\sqrt{\frac{T_c}{T}-1}\right)$ with $\alpha = 1.138$ determined from fitting $\Delta(T)$ to the temperature dependence of the gap magnitude obtained from the BCS gap equation (Supplementary Figure 5a). Using $\Delta_0 = 1.764 k_B T_c$ reproduces the BCS behaviour, as shown in Supplementary Figure 5b.

Supplementary References

[1] Ionov, V. M. *et al.* Anisotropy of physical properties and crystal structure of PdBi in the interval 293 − 570K. *Sov. Phys. Crystallogr.* **34**, 496–499 (1989).

[2] Joshi, B., Thamizhavel, A. & Ramakrishnan, S. Superconductivity in noncentrosymmetric BiPd. *Phys. Rev. B* **84**, 064518 (2011).

[3] Mühlschlegel, B. Die thermodynamischen Funktionen des Supraleiters. *Zeitschr. Phys.* **155**, 313–327 (1959).

[4] Macdonald, A. H., Pickett, W. E. & Koelling, D. D. A linearized relativistic augmented-plane-wave method utilizing approximate pure spin basis functions. *J. Phys. C* **13**, 2675–2683 (1980).

[5] Joshi, B., Thamizhavel, A. & Ramakrishnan, S. Probing anisotropy in a new noncentrosymmetric superconductor BiPd. *JPS Conf. Proc.* **3**, 015010 (2014).
